# Online psychological intervention with LGBT clients in Portugal

**DOI:** 10.1192/j.eurpsy.2021.925

**Published:** 2021-08-13

**Authors:** H. Pereira, M. Figueira

**Affiliations:** 1 Ubi, Research Centre in Sports Sciences, Health Sciences and Human Development, Covilha, Portugal; 2 Psychology And Education, University of Beira Interior, Covilha, Portugal; 3 Cics-ubi, Centre for Research in Health Sciences, Covilha, Portugal

**Keywords:** Depression, Anxiety, LGBT, Online intervention

## Abstract

**Introduction:**

This is a quasi-experimental and pioneering study in Portugal.

**Objectives:**

(1) to provide assessment materials for symptoms of internalized homophobia, depression, and anxiety targeted at LGBT people; (2) offer support materials for psychotherapeutic work-oriented in the areas of internalized homonegativity, depression, and anxiety; and (3) offer monitoring measures throughout the program to demonstrate changes. It consists of three phases (pre-program evaluation, therapeutic activities and post-program evaluation).

**Methods:**

38 LGBT + individuals participated, average age was 34.15 years, 30 self-identified as male. Measures used for the pre and post-intervention assessment were the sociodemographic questionnaire, the LGBT identity questionnaire, the Rosenberg self-esteem scale, and the BSI-18. Participants were invited to join the program online, through a platform created for this purpose, where ethical aspects were clarified, namely: confidentiality and commitment to adherence. Therapeutic tasks were sent by email or WhatsApp depending on the preference of each participant.

**Results:**

Relevant differences in internalized homophobia, depressive, and anxious symptoms between the pre and post-intervention moments were observed, indicating that the program is effective in changing these symptoms.

**Conclusions:**

The importance of validating this type of program allows reaching “hidden” populations by offering online support that minimizes the effects of sexual stigma on LGBT + populations.

